# A molecular conveyor belt-associated protein controls the rotational direction of the bacterial type 9 secretion system

**DOI:** 10.1128/mbio.01125-25

**Published:** 2025-06-13

**Authors:** Abhishek Trivedi, Jacob A. Miratsky, Emma C. Henderson, Abhishek Singharoy, Abhishek Shrivastava

**Affiliations:** 1 School of Life Sciences, Arizona State University 69991, Tempe, Arizona, USA; 2 Biodesign Institute, Arizona State University 43363, Tempe, Arizona, USA; 3 Center for Biological Physics, Arizona State University 7864, Tempe, Arizona, USA; 4 School of Molecular Sciences, Center for Applied Structural Discovery, Arizona State University 7864, Tempe, Arizona, USA; Indiana University Bloomington, Bloomington, Indiana, USA

**Keywords:** bacterial protein secretion, type 9 secretion system (T9SS), molecular motors, bacterial motility, gliding motility, *Flavobacterium*, chemotaxis

## Abstract

Many bacteria utilize the type 9 secretion system (T9SS) for gliding motility, surface colonization, and pathogenesis. This dual-function motor supports both gliding motility and protein secretion, where rotation of the T9SS plays a central role. Fueled by the energy of the stored proton motive force and transmitted through the torque of membrane-anchored stator units, the rotary T9SS propels an adhesin-coated conveyor belt along the bacterial outer membrane like a molecular snowmobile, thereby enabling gliding motion. However, the mechanisms controlling the rotational direction and gliding motility of T9SS remain elusive. Shedding light on this mechanism, we find that in the gliding bacterium *Flavobacterium johnsoniae*, deletion of the C-terminus of the conveyor belt-associated protein GldJ controls and, in fact, reverses the rotational direction of T9SS from counterclockwise (CCW) to clockwise (CW). This suggests that the interface between the conveyor belt-associated protein GldJ and the T9SS ring protein GldK plays an important role in controlling the directionality of T9SS, potentially by modulating its interaction with the stator complex GldLM, which drives motor rotation. Combined with MD simulation of the T9SS stator units GldLM, we suggest a “tri-component gearset” model where GldJ controls the rotational direction of its driver, the T9SS, thus providing adaptive sensory feedback to influence the motility of the gliding bacterium.

**IMPORTANCE:**

The type 9 secretion system (T9SS) is fundamental to bacterial gliding motility, pathogenesis, and surface colonization. Our findings reveal that the C-terminal region of the conveyor belt-associated protein GldJ functions as a molecular switch which is capable of reversing the rotational direction of T9SS. Through the coordinated actions of the T9SS stator units (akin to a driving motor), the GldK ring (the gear that converts rotational energy into linear movement), and GldJ, this machinery forms a smart conveyor belt system reminiscent of flexible or cognitive mechanical conveyors. Such advanced conveyors can alter their direction to adapt to shifting demands. Here, we show that the bacterial T9SS similarly adjusts its rotational bias based on feedback from the conveyor belt-associated protein GldJ. This dual-role feedback mechanism underscores an evolved, controllable biological snowmobile, offering new avenues for studying how bacteria fine-tune motility in dynamic environments.

## INTRODUCTION

To date, three classes of ion-driven biological rotary motors have been identified: the type 9 secretion system (T9SS), ATP synthase, and the bacterial flagellar motor (1). The T9SS, discovered recently, is utilized by the Bacteroidetes–Chlorobi–Fibrobacteres superphylum bacteria. Around 300,000 distinct proteins secreted by the T9SS have been cataloged in InterPro (2). These include, but are not limited to, motility adhesins, proteases, and enzymes that degrade complex polysaccharides (3). Changes in abundances of T9SS-encoding Bacteroidetes in human microbiota correlate with several diseases, and the T9SS of a gut Bacteroidetes, *Paraprevotella clara*, plays a crucial role in defending against viral infections (4). In contrast, in the case of oral microbiota, the inflammation induced by T9SS-secreted gingipain proteases of *Porphyromonas gingivalis* is associated with the onset of periodontitis and Alzheimer’s disease (5–7). Furthermore, in some cases, T9SS is required for the development of virulent biofilms (8–10), which are harmful to a variety of hosts, including humans (6), birds (11), and fish (12, 13).

**Fig 1:**
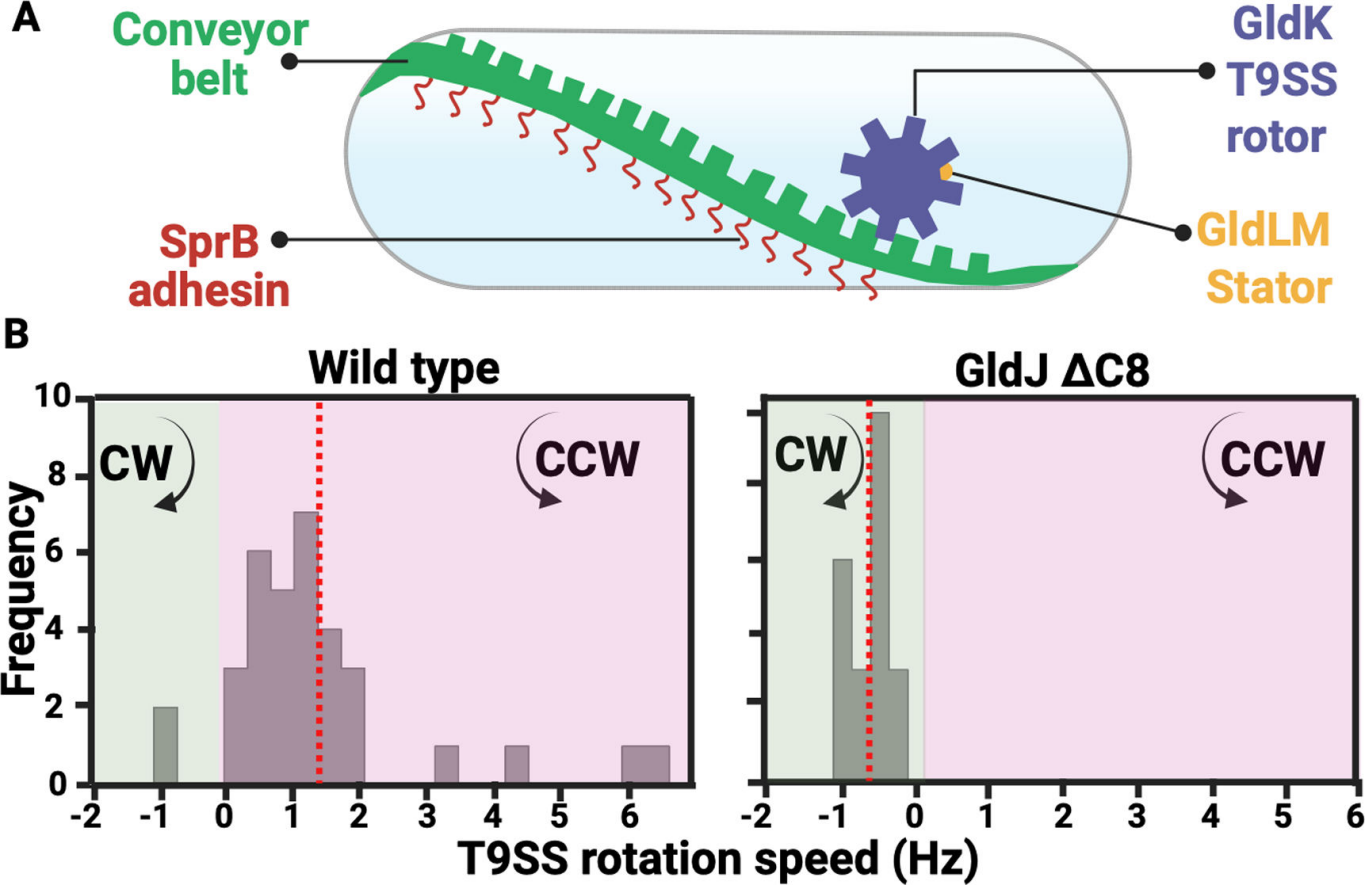
The C-terminal region of a conveyor belt-associated protein, GldJ, controls the rotational direction of T9SS. (**A**) A cartoon showing the current rack and pinion model, where the GldK ring of rotary T9SS (pinion) drives the GldJ, part of conveyor belt (rack). (**B**) Frequency distribution of rotational speed shows that the outer portion of the T9SS in wild-type cells (*n* = 33) rotates primarily in the counterclockwise (CCW) direction with a mean speed of 1.3 Hz, whereas T9SS of cells lacking the C-terminal region of GldJ (GldJ ΔC8, *n* = 21) (CJ2443) rotates in the clockwise (CW) direction with a mean speed of 0.6 Hz. The red dotted line represents the mean rotational speed, and the + and − signs indicate CCW and CW rotational directions, respectively.

The T9SS also enables the gliding motility of Bacteroidetes over human and animal tissues, fish scales, sediments, and plant roots (14–16). *Flavobacterium johnsoniae*, found in the soybean rhizosphere (17) and aquatic ecosystems, is a model organism used for the investigation of both the macromolecular mechanics of T9SS and the gliding motility of Bacteroidetes. The T9SS rotates in place around a stationary axis, and its rotational motion is driven by a proton motive force (1). Following measurements of fluorescently labeled T9SS, tracking of motility adhesins on the conveyor belt, and tethered cell analysis, it has been suggested that, similar to a macromolecular rack and pinion assembly, the T9SS propels a polymeric cell-surface conveyor belt over the cell body, thus mobilizing a molecular snowmobile that enables the gliding motility of Bacteroidetes (18) (Fig. 1A). Generally, the T9SS stands out as a multifunctional apparatus. It is known to provide a competitive advantage to a cell by converting the energy of ionic flow into rotational motion, with the generated rotational energy being utilized to enable either secretion processes or direct mechanical coupling with a conveyor belt, thus facilitating cellular locomotion. Although gliding motility represents a distinctive example of cellular evolution, nothing is known about molecular mechanisms regulating rotational direction or the mechanical linkage between T9SS and the conveyor belt. In *F. johnsoniae*, known for its robust gliding, the rotation of the GldKN ring (a GldK ring stacked on top of a GldN ring) is thought to propel the linear motion of a cell-surface adhesin, SprB, along a conveyor belt that is associated with the protein GldJ (19–21). The direct surface interaction between SprB and an external substratum enables gliding motility (19). Intriguingly, GldJ is missing from T9SSs in nonmotile bacteria, such as *P. gingivalis*, which utilize T9SS exclusively for protein secretion and pathogenesis (5).

The T9SS consists of around 20 unique proteins working together to enable both gliding motility and protein secretion (22). These building blocks do not exhibit sequence homology with the extensively studied bacterial flagellar motor that enables swimming motility of other bacteria. The mechanical motion in T9SS is enabled by proton-conducting GldLM stator units exhibiting 5:2 structural stoichiometry. A large portion of GldL is cytoplasmic with transmembrane helices that form a pentameric cage enclosing the transmembrane portion of a periplasmic GldM dimer. GldL localizes on the rotational axis of tethered cells, and in gliding cells, it remains stationary with respect to the cell body (23, 24). The GldM (PorM) periplasmic arm has 18-fold symmetry, and it interacts with the outer membrane-embedded GldKN (PorKN) ring (25). The macromolecular structure and 5:2 stoichiometry of GldLM are reminiscent of the flagellar MotAB proteins, suggesting similar principles in their evolution. The T9SS and bacterial flagellar motor both use ion channels to spin a ring, but the location of the ring differs. Specifically, in the bacterial flagellar motor, proton-conducting MotAB stator units drive the rotational motion of a cytoplasmic ring (C-ring), and the rotational bias is controlled by the cytoplasmic sensory transduction protein, CheY-P, which binds to the C-ring (26, 27). In contrast, the GldLM-driven rotation of the GldKN ring of T9SS occurs in the outer membrane. Extending the logic that allosteric changes in its C-ring determine the rotational direction in bacterial flagellar motor, it could be hypothesized that some protein binding to the GldKN ring might regulate the rotational bias of T9SS. This suggests the presence of an outer membrane-associated sensory transducer, but any evidence is currently absent.

The gliding motility of Bacteroidetes showcases seamless coordination of multiple macromolecular units that drive directed cellular motion, yet its actual macromolecular mechanisms have long remained enigmatic (28, 29). Here, we find that an unlikely candidate, namely, the cell-surface conveyor belt-associated protein GldJ, controls the rotational direction of T9SS. Deletion of eight amino acids from the C-terminus of the conveyor belt-associated protein GldJ (GldJ ∆C8) switches T9SS from CCW to CW direction. Furthermore, suppressor mutagenesis revealed mutations in the T9SS ring protein GldK that partially complemented the loss of GldJ’s C-terminal region, suggesting conservation of interaction energy between the T9SS ring protein GldK and the conveyor belt-associated protein GldJ. The T9SS motor is driven by the direct interaction of individual GldM proteins within GldLM stator units with the GldKN ring (23). Unraveling the dynamics of the stator, our steered and Bayesian molecular dynamics (MD) simulations suggest a CW rotational bias for GldM stator, and we do not find any suppressor mutants in GldLM, suggesting that the directionality of T9SS is controlled at the GldK–GldJ interface. Together, our results here suggest that GldLM stator units, GldKN ring, and the GldJ-associated conveyor belt constitute a tri-component gearset, driving the gliding motility in Bacteroidetes. Also, the stator complex harnesses proton motive force to propel the GldKN ring which, in its turn, facilitates and controls the mechanical motion of the conveyor belt-associated protein GldJ. Our model suggests a unique molecular mechanism that alters the directionality of cellular motility, whereby a component of the cell-surface conveyor belt (GldJ) enables a regulatory feedback loop influencing the rotational bias of its associated gear (GldKN ring) and thereby altering the directionality of the propulsive motion of the conveyor belt.

## RESULTS

### The conveyor belt-associated protein GldJ controls directionality of T9SS motor

It has been reported that, in wild-type *F. johnsoniae*, 90% of T9SS units rotate CCW, whereas the remaining 10% rotate CW (1). Using an established assay for the T9SS rotation (1), in which viscous shear is applied to potentially disrupt the conveyor belt and cells are tethered to a glass surface with an anti-SprB antibody, we collected additional data to quantify both the speed and direction of the T9SS motors. In agreement with earlier findings, our data confirm that the T9SS rotates bidirectionally with a bias toward CCW direction (Fig. 1B; Video S1). Functional T9SS motors are essential for the motion of the cell-surface conveyor belt (30, 31). Deletion of the conveyor belt-associated protein GldJ destabilizes GldK, and cellular levels of GldK are significantly reduced (31). Yet, when eight specific amino acids on the C-terminus of GldJ are deleted (GldJ ΔC8), GldK levels are maintained. However, despite near-wild-type levels of GldK, GldJ ΔC8 cells do not exhibit swarming motility on agar (31). These observations suggest that GldJ might somehow influence the rotation or assembly of T9SS. To test this conjecture, GldJ ΔC8 cells were sheared and tethered to a glass surface. Surprisingly, our experimental findings revealed a distinct change in T9SS rotational directionality as tethered T9SS motors rotated exclusively CW in GldJ ΔC8 cells (Fig. 1B; Video S2). In wild-type cells, T9SS exhibited an average rotational speed of 1.3 Hz (CCW), whereas in GldJ ΔC8 cells, the rotational speed was about 0.6 Hz (CW; Fig. 1B). The above results indicate that the eight C-terminal amino acids of GldJ play a crucial role in regulating both the speed and directionality of the T9SS motor. In the absence of those amino acids of the conveyor belt-associated protein GldJ, T9SS rotates exclusively CW.

### Suppressor mutagenesis revealed that point mutations within GldK in GldJ ∆C8 cells restored swarming

**Fig 2:**
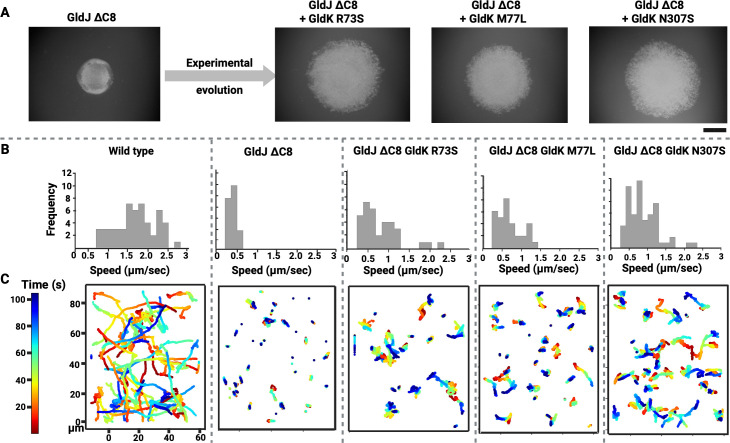
Experimental evolution restored gliding motility in cells that lack the C-terminal region of GldJ. (**A**) Cells lacking the C-terminal region of GldJ (GldJ ΔC8) do not swarm on agar. Experimental evolution of the GldJ ΔC8 strain (CJ2443) resulted in partial restoration of swarming. Spontaneous mutations that restored motility in the evolved strains were in the T9SS ring protein GldK. Scale bar = 6 mm. (**B**) Frequency distribution of gliding speed (μm/sec) for wild-type and mutant cells on a glass surface. The average gliding speeds for wild-type, GldJ ΔC8, GldK R73S GldJ ∆C8 (FJASU_21), M77L + GldJ ∆C8 (FJASU_20), and N307S + GldJ ∆C8 (FJASU_19) cells were 2 µm/s, 0.4 µm/s, 0.89 µm/s, 0.64 µm/s, and 0.88 µm/s, respectively. (**C**) Trajectories of wild-type and mutant cells gliding over a glass surface (*n* > 30), color-coded by time (seconds). The average farthest displacement for cells of wild-type, GldJ ∆C8, GldK R73S + GldJ ∆C8, M77L + GldJ ∆C8, and N307S + GldJ ∆C8 strains was 27 µm, 1.79 µm, 6.23 µm, 4.87 µm, and 8.14 µm, respectively.

Wild-type *F. johnsoniae* swarms on PY2 agar, whereas GldJ ΔC8 cells do not (31). Suppressor mutagenesis of GldJ ΔC8 cells yielded strains that exhibited improved swarming compared to the parent GldJ ΔC8 strain. (Fig. 2A; Fig. S1). Three individual suppressor strains were isolated, and their whole genome was sequenced. All three had point mutations in the T9SS ring protein GldK, with the mutations occurring at the R73S, N307S, and M77L sites, respectively, which further supports the suggestion that GldJ and GldK interact closely with each other (21).

Single-cell motility over a glass surface was recorded for the wild-type, GldJ ΔC8 strain, and the GldK suppressor strains, and individual trajectories of cellular motion were plotted. Wild-type cells exhibited long runs followed by relatively quick turning events characterized by either back-and-forth cellular motion or rare head-on flips, whereas GldJ ΔC8 cells mostly exhibited minimal back-and-forth cellular motion. Cells of wild-type and GldJ ΔC8 strains exhibited average locomotion velocities of about 2 µm/s and 0.4 µm/s, respectively, and the average farthest displacement travelled by wild-type cells was 27 µm, while GldJ ΔC8 cells was 1.8 µm (Fig. 2B). It is possible that in the GldJ ∆C8 cells, the conveyor belt may either be shortened or even depolymerized. In contrast, GldK R73S, M77L, and N307S suppressor mutants of the GldJ ΔC8 background exhibited runs of reduced length, with average locomotion velocities calculated from >30 cells per strain being approximately 0.89, 0.64, and 0.88 µm/s, respectively. Notably, the oscillatory back-and-forth motion was present along with the runs. The longest runs among the three evolved GldJ ΔC8 strains occurred within the N307S population, and the average farthest displacement for GldK R73S, M77L, and N307S cells was 6.2 µm, 4.9 µm, and 8.1 µm, respectively (Fig. 2C; Video S3). This implies that point mutations in the T9SS rotor protein GldK, which has around 30% sequence similarity to GldJ (Fig. S1B), partially restored the mostly nonoperational conveyor belt of the parent GldJ ∆C8 strain.

**Fig 3:**
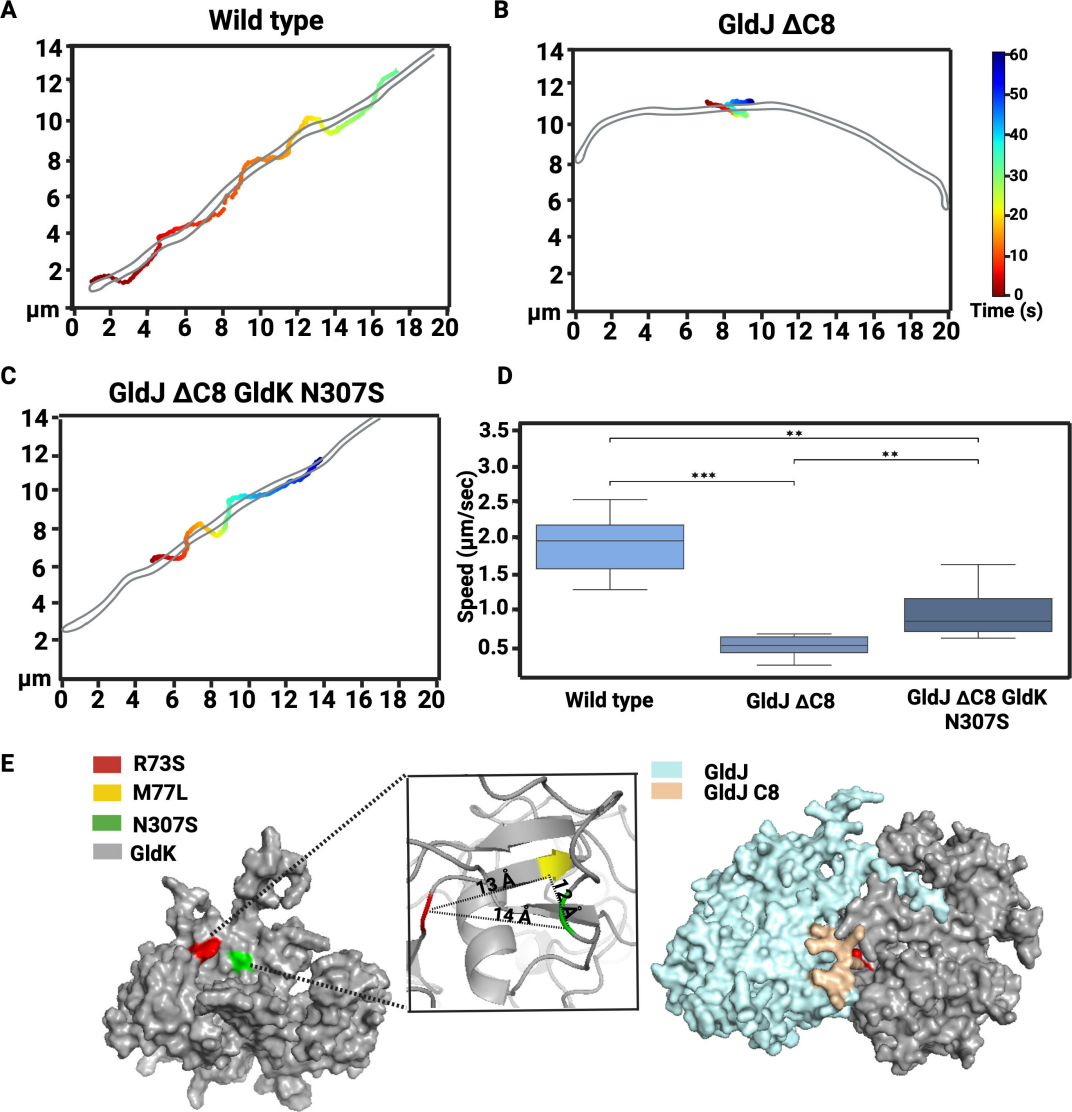
Motion of the cell-surface adhesin SprB is partially restored in GldK N307S carrying GldJ ΔC-terminal cells. (**A through C**) A representative trajectory of fluorescently labeled SprB moving along the conveyor belt of wild-type and mutant cell (FJASU_19). (**D**) A box plot showing speeds of at least six SprB molecules for each strain. Statistical significance was analyzed via two-tailed Mann–Whitney test. *P* value: *P < 0.05, 0.001 < **P ≤ 0.01, ***P < 0.001. (**E**) Predicted structure of GldK showing the proximity of R73, M77, and N307 identified via the suppressor screen. In the surface representation, M77 is not visible, but the cartoon view (inset) displays all three residues. Docking of the conveyor belt-associated protein GldJ (cyan) with the T9SS rotor protein GldK (gray) shows that the GldJ C8 region fits into a GldK cleft region that contains R73 and N307 with M77 in the vicinity.

To gather more information on the integrity of the conveyor belt, the mobile cell-surface adhesin, SprB, which is attached to the conveyor belt, was fluorescently labeled, and its movement was tracked. Trajectory of a single SprB signal provided information about the structural integrity of the conveyor belt. As anticipated, cephalexin-treated elongated wild-type cells (20) exhibited long trajectories with SprB moving at about 2 µm/s, and they appeared to have fully formed, closed conveyor belts looping around cell poles. In contrast, the GldJ ΔC8 strain exhibited drastically short SprB trajectories characteristic of the oscillatory motion at about 0.5 µm/s, suggesting either immobilization or depolymerization of the conveyor belt. However, the N307S strain of the GldJ ΔC8 background exhibited longer trajectories akin to the wild type. This indicates that the functionality of the conveyor belt was restored, at least partially, because of the abovementioned point mutations in the GldK ring of T9SS. In the latter case, the velocity of SprB was about 1 µm/s which about half of the SprB speed of wild-type cells—suggesting that the T9SS rotor to conveyor belt interaction was partially restored (Fig. 3; Video S4). To summarize, swarming, single-cell motility, and SprB tracking provided evidence that the interaction between the C-terminal region of GldJ and the three experimentally identified mutations of GldK might be crucial for maintaining proper operation of the conveyor belt.

Additionally, the three GldK suppressor strains isolated from our screen also had an N178K mutation in a putative LuxR-like protein (*Fjoh_4220*) and a G223V mutation in an acyl carrier protein (ACP) synthase (*Fjoh_3810*). A detailed genetic, single-cell motility, and swarming analysis showed that *fjoh_4220* and *fjoh_3810* do not impact gliding motility (Fig. S2 and S3, supplemental text, and Videos S5 to S8).

### Suppressor mutagenesis unlocked some of the CW-only T9SS motors

As evident by the data presented in Fig. 1, T9SS of the parent GldJ ∆C8 strain is “locked” in the CW-only rotational state. Because the evolved GldK point mutations partially restored cellular motility lost due to ∆C8 in GldJ (Fig. 2 and 3), we tested if some of the T9SS motors in the evolved strains could rotate CCW. Indeed, the GldK point mutations influenced the rotational bias, with some T9SS motors of the evolved strains rotating CCW, albeit about 5–10 times slower than those having wild-type GldK. At such low rotational frequencies, it is difficult to differentiate between rotational motion and Brownian fluctuations. Therefore, only the faster population of T9SS motors that exhibited at least one full revolution was further analyzed. The above criterion increased the significance of our results, but, simultaneously, it reduced the overall population of T9SS motors that were subjected to the analysis. We found that in the R73S strain of GldJ ΔC8 background, one out of six T9SS motors taken into consideration rotated CCW (Fig. S4). In the N307S strain, the above was true for one out of 16 T9SS motors (Video S9). Contrary to the wild-type motors that exhibit CCW bias, T9SS of the evolved strains retained the CW bias of the parent strain. However, in both the wild-type and experimentally evolved strains, cellular motility was observed irrespective of the direction of the rotational bias (Fig. 2). In both cases, a rotational bias (whether CCW or CW) of about 85% to 95%, with approximately 5% to 15% of motors rotating in the opposite direction (noise), was associated with long runs that are characteristic of typical gliding trajectories. In the absence of noise, i.e., 100% CW bias characteristic of the GldJ ΔC8 strain, the cellular motility was diminished (Fig. 1B and 2C).

A gliding cell is equipped with about eight to 12 T9SS units rotating in unison (24, 30). If one of the units was to rotate in the opposite direction, it would lead to about 8% to 12.5% to the motors rotating in opposite direction (noise), which is in agreement with the CCW:CW bias observed in our tethered cell data (Fig. 1B). Assuming that multiple T9SS motors control a single conveyor belt, a random change in the rotational direction of any motor, due to inherent noise, could lead to slipping of the conveyor belt. This slippage might then cause disorientation and potentially change the gliding direction of the cell. Within seconds, the slipped conveyor belt might diffuse and re-engage with T9SS motors rotating concertedly, leading to the restoration of smooth gliding behavior, albeit in a new direction. The observation that gliding cells follow straight trajectories interspersed with sudden turning events (Fig. 2C) supports the mechanism suggested above. In their natural environments, gliding cells release enzymes to decompose complex polysaccharides, such as chitin and cellulose found in insoluble food sources (32, 33). Their long gliding movements, interspersed with sudden turns, may enhance their ability to locate and digest these food sources. This behavior suggests evolutionary pressure for a strong CCW rotational bias, with significant CW rotation. In the lab, we have observed this pattern in wild-type strains (Fig. 1B and 2C) and its reversal in evolved strains, where a prominent CW bias is coupled with noticeable CCW rotation (Fig. S4). The observed “inverse state,” characterized by slower cell movement, suggests that the interaction energy between T9SS units and the conveyor belt-associated protein GldJ differs between wild-type and evolved strains.

A predicted AlphaFold tertiary structure of GldK shows that the residues R73, M77, and N307 are packed close together in an arrangement which resembles a cleft, with the R73 to N307, R73 to M77, and M77 to N307 inter-residue distances being 14 Å, 12 Å, and 13 Å, respectively (Fig. 3E). Furthermore, GldK has been shown to facilitate the oligomerization and complex formation of GldJ (21). Consequently, molecular docking of GldJ and GldK was performed using HDOCK (34). One of the models, which has an 86% accuracy score, indicates that the C-terminal region of GldJ fits into the cleft formed by GldK and aligns with the genetic data (Fig. 3E). Therefore, it was selected for further analysis. MD simulations performed on the docked GldJ-GldK model revealed that deleting the C8 region of GldJ causes the cleft region of GldK, which contains residues R73, M77, and N307, to become more dynamic and expand towards the area previously occupied by C8 of GldJ (Fig. S5). This explains why mutations in the ring protein GldK can compensate for the loss of the C-terminal region of the conveyor belt-associated protein GldJ. This provides further evidence that the phenotypes described above might be due to changes in the interaction energy between the T9SS ring and the conveyor belt. Interestingly, the combination of GldK mutations R73S, M77L, and N307S in a GldJ ∆C8 background does not restore either swarming on agar or single-cell motility on a glass surface (Fig. S6 and supplemental text). This suggests that these three GldK point mutations together might completely hinder the interaction of the rotor with the conveyor belt-associated protein GldJ. Any one of the three GldK mutations is sufficient to alter the function of the cleft region of GldK and, consequently, its interaction energy with GldJ ∆C8.

### Molecular simulations suggest that GldLM stator units rotate in the clockwise direction

In a gliding cell, the GldKN/PorKN ring interacts with the GldLM/PorLM stator units (35), which harness pmf to power the rotation of the T9SS (25). Recent research has demonstrated that in flagellated bacteria, the MotAB components of the bacterial flagellar motor rotate CW, driving the rotation of C-rings of the bacterial flagellar motor (36). The proton-conducting GldLM stator units have an asymmetric 5:2 structure similar to MotAB. However, despite this conceptual similarity, the actual rotational dynamics of GldLM stator units at the atomistic level have not yet been explored. Given that MotAB components rotate unidirectionally CW (36), analyzing whether GldLM stator units are bidirectional or unidirectional may help elucidate if GldLM plays any role in the directional switching of T9SS. To investigate this, we employed MD simulations to model the rotational dynamics of GldLM.

The probability of CW vs. CCW GldLM rotation was estimated via MD. Notably, GldLM has a characteristic structural asymmetry with two GldM chains distributed among five GldL subunits. The latter feature precludes uniformity of GldL–GldM interactions, and it has been suggested that a single GldM chain can only be engaged into a channel-specific ion pairing involving protonated ARG 9 of GldM and deprotonated GLU 49 of GldL at a time (23). This ion pair is implicated in transporting protons and utilization of the proton motive force. With the PDB all-atom macromolecular structure featuring GldLM symmetry mismatch as an input (see PDB: 6YS8), three different MD simulations were performed on the GldLM stator units.

**Fig 4:**
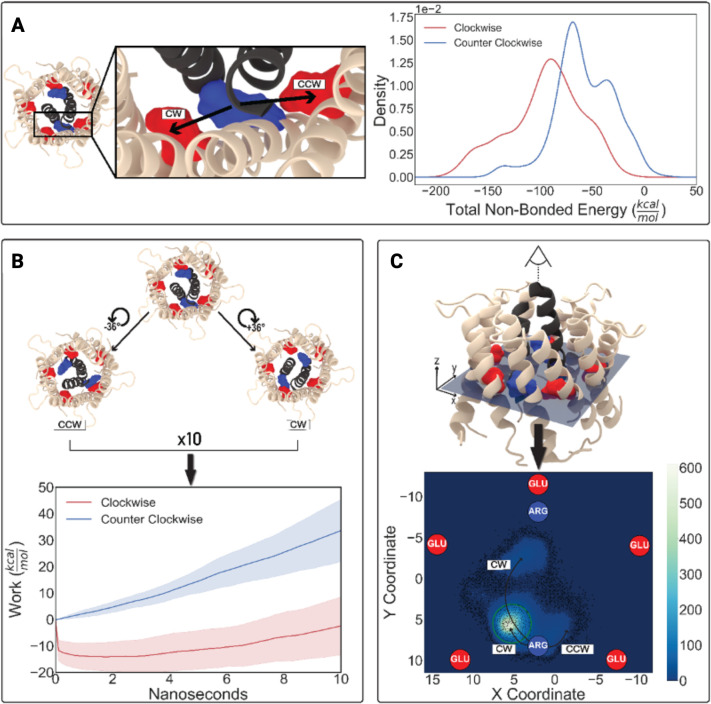
MD simulations suggest a preference for clockwise rotation of the driver gear, GldM, in the tri-component gearset. (**A**) Equilibrium MD simulations and nonbonded energy calculations show that the central arginine residue of GldM interacts more favorably with the glutamate residue of GldL in the CW direction. The kernel density estimation (KDE) method was used to estimate the probability density function of the nonbonded energy from the simulation trajectory. (**B**) Central: unaltered equilibrium configuration of the initial structure, excluding the membrane for the sake of clarity. Left: structure after a CCW GldM trans-membrane helix (TMH) rotation of −36°. Right: result after +36° CW rotation. The plot illustrates the nonequilibrium energetics from 10 consecutive SMD simulations (360° total in each direction), which reveal a lower work value for CW GldM TMH rotation. (**C**) Three-dimensional representation of the central structure shown in panel B. A top-down view highlights the plane used for the statistical analysis of post-MELD simulations. The spatial coordinates of the unbound arginine residue are mapped in a contour plot, with lighter shades indicating higher density, which suggests an unbiased preference for CW rotation. The arrows indicate the trajectory of the positional displacements of the unbound arginine. A green circle superimposed on the plot serves as the boundary where the probability of a datum falling within the enclosed region was computed.

We began with an all-atom equilibrium MD simulation where the nonbonded interaction energies were monitored across the simulation trajectory to explore the contacts between ARG 9 of GldM and GLU 49 of GldL, where GldL is either in CCW or CW orientation. We obtained the mean values of −57.5 and −94.6 kcal/mol for the CCW-oriented GLU 49 and CW-oriented GLU 49 on GldL, respectively (Fig. 4A). The rotation of transmembrane helices of GldM within GldL is inaccessible using traditional MD techniques. Hence, 20 replicas of 10 ns-long steered MD (SMD) simulations were explored. The results are indicative of a significant relationship between the average work and rotational directionality preference, suggesting a bias towards CW (Fig. 4B).

Prompted by the apparent differences in work profiles, we sought to obtain a more reliable description of the CCW vs. CW occupancies in GldM through modeling employing limited data (MELD) or MELD-guided MD simulations (37). We began with a configuration in which the ARG 9 residue was equidistant from the CCW and CW deprotonated GLU 49 of GldL to avoid any rotational bias in our model. Upon convergence, our MELD simulation revealed that around 82% of ARG tend to be spontaneously located in the immediate vicinity of the CW-oriented GLU, in the absence of any directive steering force throughout the simulation. Thus, as opposed to the (CCW biased) GldK ring of T9SS, the proton-conducting GldM–GldL transmembrane helix complex of T9SS appears to have a CW bias (Fig. 4C). In our MD simulations, GldLM ion channels rotated mostly CW, and we observed no transitioning to CCW rotation. Hence, the MD simulations suggest that the observed change in directional bias of the T9SS might not result from an altered rotational directionality of the GldLM ion channels that drive the system.

## DISCUSSION

**Fig 5:**
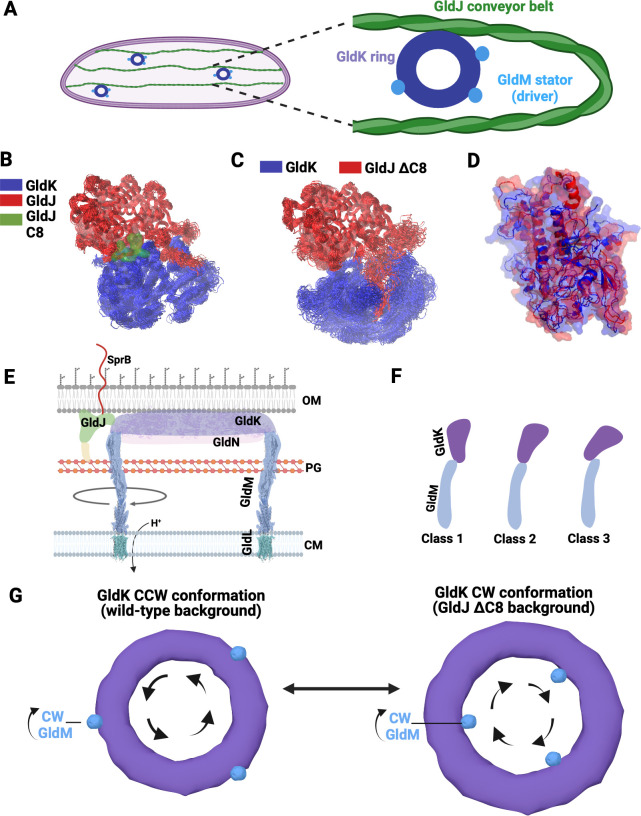
A model for directional switching of T9SS. (**A**) A cartoon showing the tri-component gearset with GldM stator unit as the driver of the T9SS ring and conveyor belt. (**B**) An overlay of frames collected for the final 50 ns of the MD simulation for the wild-type GldJ (red) and GldK (blue) system with the GldJ C8 region highlighted in green. (**C**) Upon deletion of the C8 region of GldJ, an appreciable expansion of GldK is observed in contrast to the wild-type system shown in panel B. (**D**) An overlay of the GldK from the wild-type and GldJ∆C8 systems depicts conformational changes that are >1 nm. (**E**) A cross-sectional cartoon of the gliding machinery shows the proton motive force (pmf) powered driver gear, GldM, rotating unidirectionally clockwise (CW) and pushing the T9SS ring. This action results in the movement of the GldJ and SprB adhesin either towards the viewer or away from the current image plane. Images are not to scale. (**F**) A cartoon summarizing the cross-section of different conformational states (class 1, 2, and 3) of T9SS ring as observed by cryo-ET (25). (**G**) A top-down view of class 1 and class 3 T9SS motors as observed by cryo-ET (25) supports a physical model of T9SS directional switching. When the wild-type conveyor belt-associated protein GldJ interacts strongly with the GldK ring, T9SS maintains a conformation where GldM pushes the outer periphery of the GldK ring, causing T9SS to rotate CCW. Conversely, when the interaction energy between GldJ and GldK is reduced (GldJ∆C8), the GldK ring changes conformation. Consequently, GldM pushes near the inner periphery of the GldK ring, resulting in CW rotation of the T9SS.

Our genetic analyses, cell tethering assays, and MD simulations, combined with prior biochemical and structural data on T9SS and associated motility proteins (21, 38, 39), suggest that the T9SS GldKN ring, the GldJ-associated conveyor belt, and the GldLM stator units function as three interlocking mechanical gears. We term this interaction a tri-component gearset. In this system, the GldLM stator unit serves as the primary gear and driver, utilizing proton motive force. The stator unit drives the rotation of the middle gear, the GldK ring, which in turn propels the outer component, the conveyor belt. Cryo-ET has revealed that the conveyor belt-associated protein GldJ forms a closed loop, with multiple loops observed within a single cell (21). When examined in the 2D plane, the outer loops of the tri-component gearset appear as linear conveyor belts (Fig. 5A). Our findings suggest that the conveyor belt-associated protein GldJ influences the rotational directionality of the GldK-containing T9SS ring.

Here, we advanced a working model by estimating the interaction energy between GldJ and GldK monomers using MD simulations. The simulation trajectories were analyzed to assess van der Waals and electrostatic interactions between GldJ and GldK at every timestep over a 100 ns simulation period. For the monomers, the total nonbonded GldJ-GldK interaction energy decreased by about 25% upon removal of the eight C-terminal amino acids from GldJ (Fig. S7). We observed a significant conformational change in the tertiary structure of monomeric GldK upon interaction with GldJ∆C8 compared to wild-type GldJ (Fig. 5B and C). Additionally, root mean square deviation (RMSD) values for GldK reformations increased by 10 Å after the interaction interface with GldJ was altered by the C8 deletion (Fig. S8).

In the docked model, the distance between the centers of mass of GldK and both forms of GldJ increased by 7.5 Å during the MD simulation (Fig. S9), suggesting that the tertiary structure of GldK undergoes conformational changes after the deletion of the C8 region of GldJ. In contrast, the structural changes in GldJ lacking the eight C-terminal amino acids are similar to those in wild-type GldJ (Fig. S10), thus providing an internal control for the MD simulations. An overlay of GldK monomer from the GldJ∆C8 model with the wild-type GldK monomer shows several regions in which the conformation of GldK changes >1 nm (Fig. 5D). Consistent with the predictions of our simulations, recent cryo-ET supports the structural flexibility of the T9SS and shows that the GldKN (PorKN) ring of *P. gingivalis* adopts three distinct conformational classes, forming angles of 90°, 70°, and 50° with the outer membrane (25) (Fig. 5F).

Building on structural insights from *P. gingivalis* and extending our simulations to a larger scale, we propose a model where the interaction energy between GldJ and GldK may affect the size of the GldK ring, prompting a conformational shift once the C8 segment of GldJ is removed. According to our model, the rotational direction of the GldK ring (CW or CCW) depends on the interaction site of the CW-rotating GldLM stator units. When the GldLM stators engage near the outer edge of the GldKN ring, the ring rotates CCW. Conversely, when they interact near the inner edge, the ring rotates CW. This model explains our experimental data, where in wild-type cells, the majority of the T9SS motors rotate CCW, while in GldJ∆C8 cells, they rotate exclusively CW. Alternatively, it is also possible that the bend of GldM could be altered by a reduction of interaction energy between GldJ and GldK, which might lead to a change in its interaction site with the GldK ring as observed by cryo-ET (25). Our model integrates existing knowledge and establishes a foundation for future studies aimed at elucidating the multimeric structure of the gliding machinery, capturing higher-resolution views of the polymeric conveyor belt, and conducting detailed biophysical analyses of T9SS rotation in various mutants, thereby enabling the development of more robust and comprehensive models. At a cursory glance, the suggested mechanism behind directional switching of T9SS presented here seems similar to how the rotation direction of the bacterial flagellar motor is regulated (36, 40). While there is structural resemblance between the transmembrane domains of GldLM and MotAB, the proteins composing the T9SS and gliding machinery bear no sequence resemblance to those forming the bacterial flagellar motor, indicating an instance of convergent evolution.

Overall, our findings suggest that the conveyor belt-associated protein GldJ plays a dual role. In addition to being passively driven by forces exerted on it by the GldK gear, it also feeds back into the system to influence and control the rotational directionality of its driving gear. In traditional mechanical conveyor systems, motors and gears propel the belt along fixed paths, while advanced flexible or cognitive conveyors are equipped with routing algorithms that dynamically adjust the direction based on changing requirements (41). Shaped by evolutionary pressures, the bacterial gliding machinery described here may represent a unique example of a conveyor belt equipped with sensory feedback. The data presented here suggest that bacteria have evolved a smart conveyor belt system capable of reciprocally altering the rotational bias of its driver, thereby potentially functioning as a dynamic and adaptive sensor or controller. Our data provide a basis for future studies into the molecular mechanisms and adaptive responses of gliding bacterial cells, whose inner workings appear to resemble a controllable biological snowmobile.

## MATERIALS AND METHODS

### Strains, media, and culture conditions

For standard culture maintenance, genetics, and tethered cell analysis, wild-type *F. johnsoniae* CJ 1827 (42) cells were grown in casitone yeast extract (CYE) broth (10 g casitone per liter, 5 g yeast extract per liter, 1 M Tris-HCl, pH 7.5) at 30°C with 1.5% agar. *Escherichia coli* cells used for preparation of genetic constructs were grown in Luria Bertani medium (10 g Tryptone per liter, 10 g NaCl per liter, and 5 g yeast extract) at 37°C. Unless otherwise noted, antibiotics were used at the following concentrations: ampicillin (100 µg/mL), erythromycin (100 µg/mL), streptomycin (100 µg/mL), and cephalexin (75 µM or 26 µg/mL).

### Suppressor mutagenesis

*F. johnsoniae* strains lacking eight amino acids from the C-terminal of GldJ (GldJ553/CJ2443) were grown in MM at 30°C for 24 hours. A volume of 20 µL was taken and spotted at the center of 20 different PY2 plates, dried for 20 min, and kept for incubation (lid up) in a 100% relative humidity chamber at 25°C for 3–4 days. Spreading colonies flared out on PY2 plates (Fig. S1). These flares were streaked for isolation, and single spreading colonies were selected. Genomic DNA was isolated using the GeneJET Genomic DNA Purification Kit (K0722), and the whole genome was sequenced. As a result, three individual strains, which had the following mutations—GldK R73S (strain name: FJASU1), GldK N307S (strain name: FJASU3), and GldK M77L (strain name: FJASU4)—flared out as suppressor mutants from (GldJ∆C8/CJ2443). All spreading colonies also had the G223V mutation in ACPsynthase (*fjoh_3810*) and N178K mutation in putative LuxR (*fjoh_4220*). Separate mutagenesis of each gene revealed that *fjoh_3810* and *fjoh_4220* did not influence this phenotype (Fig. S2 and S3).

### Construction of point mutations

To obtain the 2 kb fragment of *gldK* from the suppressor mutants FJASU1, FJASU3, and FJASU4, Phusion high-fidelity PCR master mix (Thermo Scientific) was used with primers PASU52 and PASU53. The resulting fragment was then digested with BamHI and SalI restriction enzymes and cloned into the pRR51 plasmid, which had been previously digested with the same enzymes. This plasmid was then introduced into *F. johnsoniae* through triparental conjugation, and mutants were isolated as described previously (42). Briefly, a mixture of PRR51-containing *E. coli* strain, helper *E. coli* strain, and CJ1827 was spotted onto a CYE agar plate containing CaCl_2_. The next day, the mixture was plated with dilutions on a CYE plate containing erythromycin to select for cells that had undergone the first recombination step. The erythromycin-resistant colonies were cultured overnight in CYE broth and then plated with dilutions on a PY2 agar plate containing streptomycin to select for cells that had undergone the second recombination step. Point mutations in ACP and LuxR were generated via a similar method with the following exceptions—primers PASU26 and PASU27 were used to amplify the ACP synthase region, while primers PASU50 and PASU51 were used to amplify the putative LuxR region. All strains, plasmids, and primers used in this study are listed in Tables 1 and 2. All mutations were confirmed by Sanger sequencing (Azenta Life Sciences). Sequencing data are available at 10.5281/zenodo.15170610.

**TABLE 1:** Strains and plasmids

Strain or plasmid	Genotype or description^*a*^	Reference
Strains		
CJ1827	rpsL2; (Sm^r^). Wild-type strain, derived from ATCC 17,061T (UW101), utilized in the construction of the deletion mutants described below.	(42)
CJ2443	*GldJ* ΔC8	(31)
FJASU_1	*GldJ* ΔC8 *fjoh_4220* (N178K) *fjoh_3810* (G223V) *GldK* R73S.	This study
FJASU_3	*GldJ* ΔC8 *fjoh_4220 fjoh_3810 GldK* N307S.	This study
FJASU_4	*GldJ* ΔC8 *fjoh_4220 fjoh_3810 GldK* M77L.	This study
FJASU_11	*GldJ* ΔC8 *fjoh_3810* (G223V)	This study
FJASU_12	*GldJ* ΔC8 *fjoh_4220* (N178K)	This study
FJASU_13	*GldJ* ΔC8 *fjoh*_4220 (N178K) *GldK* R73S	This study
FJASU_14	*GldJ* ΔC8 *fjoh*_4220 (N178K) *GldK* N307S	This study
FJASU_15	*GldJ* ΔC8 *fjoh_*4220 (N178K) *GldK* M77L	This study
FJASU_16	*GldJ* ΔC8 *fjoh_3810* (G223V) *GldK* R73S	This study
FJASU_17	*GldJ* ΔC8 *fjoh_3810* (G223V) *GldK* N307S	This study
FJASU_18	*GldJ* ΔC8 *fjoh_3810* (G223V) *GldK* M77L	This study
FJASU_19	*GldJ* ΔC8 *GldK* N307S	This study
FJASU_20	*GldJ* ΔC8 *GldK* M77L	This study
FJASU_21	*GldJ* ΔC8 *GldK* R73S	This study
FJASU_54	*GldJ* ΔC8 *fjoh_3810* (G223V) *fjoh_4220* (N178K)	This study
FJASU_75	*GldJ* ΔC8 with *GldK* R7SS, M77L, N307S	This study
Plasmids		
pRR51	rpsL-containing suicide vector; Ap^r^ (Em^r^);	(42)
ES27	Suicide plasmid used to introduce point mutation in *fjoh_4220*; *fjoh_4220* (N178K) using pRR51.	This study
ES28	Suicide plasmid used to introduce point mutation in *fjoh_3810*; *fjoh_3810* (G223V) using pRR51.	This study
ES29	Suicide plasmid used to introduce point mutation in *GldK*; *GldK* (R73S) using pRR51.	This study
ES30	Suicide plasmid used to introduce point mutation in *GldK*; *GldK* (N307S) using pRR51.	This study
ES31	Suicide plasmid used to introduce point mutation in *GldK*; *GldK* (M77L) using pRR51.	This study
ES35	Suicide plasmid used to introduce wild-type copy of *fjoh 4220* using pRR51	This study
ES36	Suicide plasmid used to introduce wild-type copy of *fjoh_3810* using pRR51.	This study
ES37	Suicide plasmid used to introduce all three-point mutations in *GldK*; *GldK* (R73S, N307S, M77L) using pRR51.	This study

^*a*^ The antibiotic resistance phenotypes are as follows: ampicillin (Ap^r^), erythromycin (Em^r^), and streptomycin (Sm^r^). The resistance phenotypes indicated in parentheses are those expressed in *F. johnsoniae*, while those without parentheses are expressed in *E. coli.*

**TABLE 2:** Primers used in this study

Primer	Sequence 5′−3′	Used in construction of
PASU26	GCTAGGGATCCGGCACAAAGAGTTTTATTGC	ES28, BamH1 site is underlined. (GCTAG additional sequence added to all primers to improve cutting efficiency)
PASU27	GCTAGGTCGACCATGCTCCAAAGTATCCAAA	ES28, SalI site is underlined.
PASU50	GCTAGGGATCCAGATATTTTATGGCCGATGT	ES27, BamH1 site is underlined.
PASU51	GCTAGGTCGACCAGGACCAGAAAACTTAAT	ES27, SalI site is underlined.
PASU52	GCTAGGGATCCAATACATCGTTCCGCTGGAA	ES29, ES30 and ES31 BamH1 site is underlined.
PASU53	GCTAGGTCGACATTGCTTTTGCAGCTCCTTC	ES29, ES30 and ES31 SalI site is underlined.

### Measurement of swarming motility on agar

*F. johnsoniae* cells were grown in PY2 broth (2 g peptone per liter, 0.5 g yeast extract per liter, pH 7.3) overnight at 25°C with shaking at 50 rpm. Cells were then washed once with PY2 medium and adjusted to an OD_600_ of 0.1. A 2 µL sample was spotted onto the center of a 1% PY2 agar petri plate, which was then incubated at 25°C for 48 hours. After incubation, the plates were imaged using an Amscope LED Trinocular Zoom Stereo microscope (SM-2TZZ-LED-18M3) with a camera (AmScope 18MP Color CMOS, C-Mount Microscope Camera, MU1803). Three replicates were performed for each strain to assess swarming motility.

### Analysis of gliding motility on a glass surface

*F. johnsoniae* cells were grown overnight in CYE broth. Then, 50 µL of cells were inoculated in 5 mL of MM and grown for 6 hours at 25°C with shaking at 50 rpm until the OD_600_ reached 0.4. After that, 45 µL of the culture was introduced into a tunnel slide, incubated for 5 minutes, washed with 40 µL of MM, and imaged using a Nikon Optiphot phase-contrast microscope fitted with Thor Labs CMOS camera (DCC1545M). Videos were recorded at 15 fps. Custom Python scripts were used to analyze the trajectory and speed of the cells. A total of (*n* ≥ 30) cells were tracked to calculate the average speed for each strain.

### Immunofluorescence labeling of SprB and imaging via TIRF microscopy

Single colonies of the *F. johnsoniae* GldK N307S in GldJ ∆C8 strain (FJASU19) were grown overnight in MM at 25°C with shaking at 50 rpm. From the overnight saturated culture, 100 µL was used as an initial inoculum to subculture 5 mL of MM containing cephalexin and incubated at 25°C with shaking at 50 rpm, resulting in the generation of elongated cells. After 6 hours, 100 μL of culture was washed by centrifugation at 5,000 × *g* for 1 minute. The resulting pellet was resuspended in 45 µL of MM, and 5 μL of 1:10 diluted purified anti-sprB antibody was added. Anti-SprB antibody (43) was purified using Melon Gel IgG Spin Purification Kit (Thermo Scientific, 45206) as described previously (1). After incubation at room temperature for 10 min, cells were washed again via centrifugation at 5,000 × *g* for 1 minute and resuspended in 45 µL MM. The cells were treated with 2 µL of secondary antibody, Alexa Fluor 555-labeled anti-rabbit IgG (Abcam, Cambridge, UK), and incubated at 25°C for 10 minutes. The cells were washed thrice at 5,000 × *g* and were pipetted into a tunnel slide. Cells were incubated for 5 min at RT and washed gently with MM in the tunnel slide. Imaging was performed on the ONI Nanoimager microscope (Oxford Nanoimaging) equipped with 405 nm diode pump solid-state lasers. Optical magnification was provided by a Å ~ 100 oil-immersion objective (Olympus, 1.4 numerical aperture), and images were acquired using an ORCA-Flash4.0 V3 CMOS camera (Hamamatsu). TIRF images were acquired using a 20 ms exposure time, with the 561 nm photoactivation laser at 2% power. A total of at least six sprB signals were tracked for each strain, and statistical significance was analyzed via two-tailed Mann–Whitney test.

### Tethered cell assay

The cells were grown overnight in MM at 25°C with shaking at 50 rpm. From the overnight culture, 100 µL was sub-cultured into 5 mL of fresh MM and incubated at 25°C with shaking at 50 rpm. When the culture reached an OD_600_ of 0.4, cell tethering was performed as previously described (1). Briefly, a polyethylene tubing with an inner diameter of 0.58 mm was used to shear cells by passing 500 µL of the culture 50 times through the tubing using 1 mL syringes with 23 G stub adapters (Instech PE-50 tubing, Instech Luer stub LS23S, Fisherbrand sterile plastic syringe). The sheared cells were washed with MM by centrifugation at 5,000 × *g* for 1 min. A volume of 40 µL of this sample was incubated with anti-sprB antibody diluted (1:10) for 20 minutes at room temperature. After another wash at 5,000 × *g* for 1 minute in MM, the cells were pipetted into the tunnel slide. After 5 minutes, 200 μL of MM were gently flowed through the tunnel slides multiple times until residual antibody was washed away, and freely rotating cells were observed. The total number (*n*) of cells tethered for each strain was as follows: wild-type cells (*n* = 33), GldJ ΔC8 cells (*n* = 21), GldJ ΔC8 cells with the GldK N307S mutation (*n* = 17), and GldJ ΔC8 cells with the GldK R73S mutation (*n* = 6).

### Preparation of the MD model

The initial model of the membrane protein system was prepared using the CHARMM-GUI membrane builder (44–46). The protein structure, consisting of five GldL subunits and two GldM transmembrane helices (23), was uploaded in PDB format and oriented properly with respect to the membrane. A 150 x 150 Å phosphatidylcholine (POPC) bilayer was generated around the protein system followed by the addition of water molecules and a NaCl concentration of 0.15 M.

### Equilibrium MD

All-atom molecular dynamics simulations were performed using Nanoscale Molecular Dynamics (NAMD) 3.0 with the CHARMM36 and TIP3P force fields used to describe the protein, lipids, and ions (47, 48). The system was energy-minimized, followed by an explicit solvent equilibration phase under isothermal-isobaric conditions. The simulations were performed for 1 µs with 2 fs time steps. A non-bonded interaction cutoff distance of 12 Å, with a switching distance of 10 Å, was enforced. Constant temperature was maintained at 300 K utilizing the Langevin thermostat employing a damping coefficient of 1/ps (49). Constant pressure was maintained at 1 atm utilizing the Langevin barostat with the piston period and decay set to 100 fs and 50 fs respectively. Periodic boundary conditions were implemented in all dimensions. Long-range electrostatics were calculated using the particle mesh Ewald approach with 1 Å grid spacing (50).

### Steered MD

Steered molecular dynamics simulations were performed using a spin angle collective variable (CV) which measures the angle of rotation about a given axis (51). One of a two-part decomposition of a full rotation CV, the spin angle CV was used to effectively measure the angle of rotation of the GldM transmembrane helices around the *z*-axis. A total of 20 simulations were executed, evenly bifurcated into two directional categories. Each explicit solvent SMD simulation was performed for 10 ns using NAMD3 software with consistent integrator and force field parameters as well as identical ensemble conditions used during equilibrium MD. The application of an external force was implemented using a moving harmonic restraint with a force constant of 10 kcal/mol/θ². Each iterative target center was established at ±36° from its starting center. Subsequently, after 10 iterations, the GldM TMH underwent a complete rotation of ±360°. Negative magnitudes denote a counterclockwise torsional movement, whereas positive magnitudes signify a clockwise torsion.

### Modeling employing limited data (MELD)

MELD is a physics-based Bayesian computational technique that leverages OpenMM MD simulation toolkit (52). The simulation schematic involved using a 16-replica parallelization, assigning one graphics processing unit (GPU) per replica. The individual simulations each spanned a duration of 250 ns, with exchange attempts initiated at regular intervals of 10 ps. There was an implemented linear temperature range from 300 to 380 K across the replica ladder, i.e., 300 K at replica 1, 380 K at replica 16, and uniform temperature interpolation for the remaining replicas. To effectively model solvation effects in our system, we utilized the generalized Born implicit solvent model (GB-Neck2) (53).

We chose the Boltzmann distribution as the prior reflecting our belief in observing each protein conformation in the absence of external data (52). The likelihood distribution includes all external information, manifested as flat-bottom harmonic potentials, and comprised of nonnegative constraints on geometric degrees of freedom (52). The external information was incorporated from generated contact maps between selected residues. The specificities are outlined below. We applied distance constraints to the heavy atoms within GldL with an absolute confidence of 100%, ensuring the inclusion of every computed contact. We imposed identical constraints within the GldM TMH. These heavy atom constraints maintained the structural integrity of the model, especially at high-temperature replicas. There were no restraints imposed between GldM and GldL, therefore allowing flexibility and rotation. The prior and likelihood constituted the posterior distribution, which was ultimately sampled during MELD.

## Data Availability

Custom Python codes for image analysis and example data sets are freely available on our GitHub at https://github.com/Trivedi0910/A-molecular-conveyor-belt-associated-protein. The sequencing data are available on Zenodo at 10.5281/zenodo.15170610. The molecular dynamics models and additional images are available upon request. All other study data are included in the article and/or supporting information.

## SUPPLEMENTAL MATERIAL

Supplemental information mbio.01125-25-s0001.pdf Supplemental figures, text, and video legends.

Video S1 mbio.01125-25-s0002.avi Tethered wild-type cell with a counterclockwise rotating T9SS.

Video S2 mbio.01125-25-s0003.avi Tethered GldJ ∆C8 cell with a clockwise rotating T9SS.

Video S3 mbio.01125-25-s0004.avi Cells containing GldK R73S, GldK M77L, and GldK N307S individual point mutations in GldJ ∆C8 background.

Video S4 mbio.01125-25-s0005.avi Immunofluorescent SprB motion on the surface of a wild-type cell, GldJ ∆C8 cell, GldK N307S in GldJ ∆C8 background.

Video S5 mbio.01125-25-s0006.avi GldJ ∆C8 cells containing GldK R73S ACP G223V, GldK M77L ACP G223V, and GldKN307S ACP G223V.

Video S6 mbio.01125-25-s0007.avi GldJ ∆C8 cells containing GldK R73S LuxR N178K, GldK M77L LuxR N178K, and GldKN307S LuxR N178K.

Video S7 mbio.01125-25-s0008.avi Cells containing ACP_G223V and LuxR N178K mutation individually in a GldJ ∆C8 background and ACP_G223V and LuxR N178K together in a GldJ ∆C8 background.

Video S8 mbio.01125-25-s0009.avi Cells containing GldK R73S, ACPG223V, and LuxR N178K mutations in a GldJ ∆C8 background; GldK R73S, ACP G223V, and LuxR N178K mutations in a GldJ ∆C8 background; or GldK N307S, ACP G223V, and LuxR N178K mutations in a GldJ ∆C8 background.

Video S9 mbio.01125-25-s0010.avi Examples of different tethered cells of the evolved GldK mutant strain in a GldJ ∆C8 background.

ASM does not own the copyrights to Supplemental Material that may be linked to, or accessed through, an article. The authors have granted ASM a non-exclusive, world-wide license to publish the Supplemental Material files. Please contact the corresponding author directly for reuse.
