# Supplementary material for: A molecular conveyor belt-associated protein controls the rotational direction of the bacterial type 9 secretion system
Source: mBio. 2025 Jun 13;16(7):e01125-25. doi: 10.1128/mbio.01125-25 (PMC12239597; doi:10.1128/mbio.01125-25)
Supplement: Supplemental information — Supplemental figures, text, and video legends. [file mbio.01125-25-s0001.pdf]

Supplementary Information

A molecular conveyor belt associated protein controls the rotational direction of the bacterial type 9 secretion system.

Authors. Abhishek Trivedi[1-3], Jacob A. Miratsky[2,4], Emma C. Henderson[1-3], Abhishek Singharoy[2,4], Abhishek Shrivastava[1-3].

Affiliations. [1]School of Life Sciences, Arizona State University, Tempe, AZ, USA; [2]Biodesign Institute, Arizona State University, Tempe, AZ, USA; [3]Center for Biological Physics, Arizona State University, Tempe, AZ, USA; [4]School of Molecular Sciences, Center for Applied Structural Discovery, Arizona State University, Tempe, AZ, USA.

Corresponding authors. Abhishek Shrivastava. Email: ashrivastava@asu.edu; Abhishek Singharoy. Email: asinghar@asu.edu

SUPPLEMENTARY FIGURES

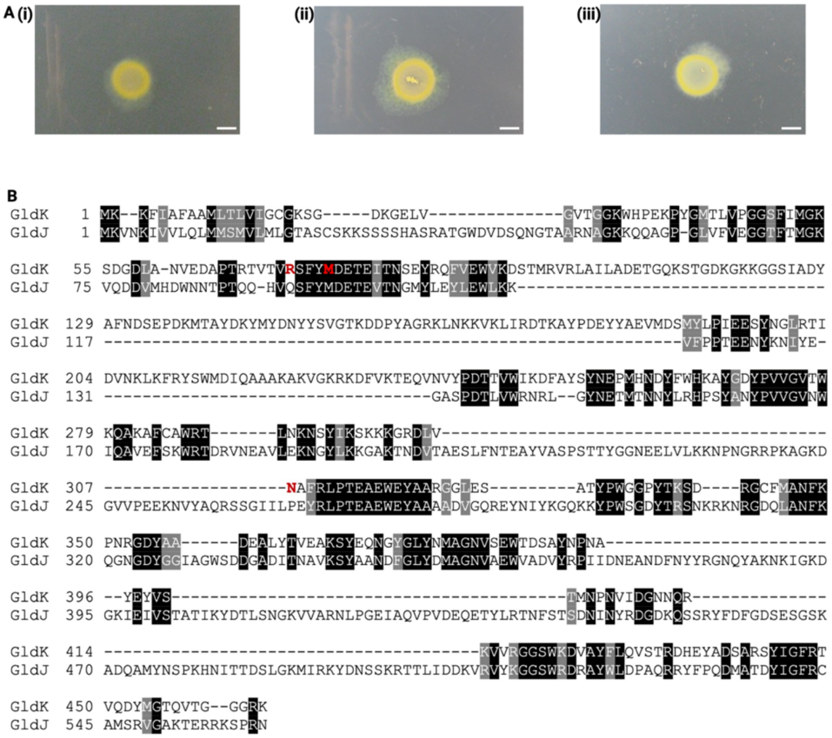

Figure S1. **(A)** Images from the experimental evolution screen of GldJ ΔC8 which lead to the following suppressors that had the ability to swarm on agar (i) GldK R73S (FJASU1) (ii) GldK N307S (FJASU3) and (iii) GldK M77L (FJASU4). Scale bar, 3 mm. **(B)** Multiple sequence alignments of *F. johnsoniae* GldJ and GldK, with residues colored in red indicating the point mutations in the GldK regions identified in the suppressor screen. These mutations restored motility in the GldJ ΔC8 mutant strain.

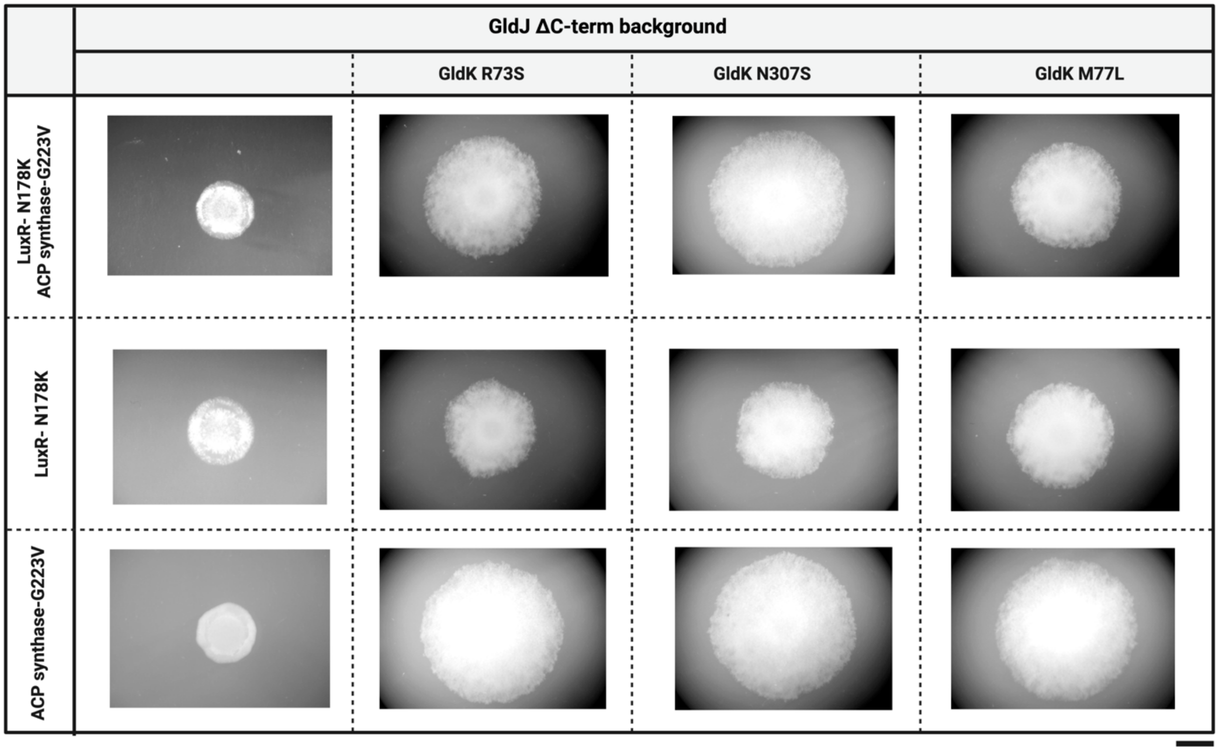

Figure S2. The evolved strains containing either GldK R73S, N307S or M77L restored the swarming motility of the parent GldJ ΔC8 (GldJ ΔC-term background) on PY2 agar plates. Scale bar, 6 mm. Whole genome sequencing revealed that evolved strains also had secondary point mutations in genes encoding an ACP synthase (G223V) and LuxR-like protein (N178K). Individual and combinatorial mutagenesis of ACP synthase and LuxR showed that these two genes do not play a role in restoration of swarming motility. Strain list from top left to right panel (FJASU_54, FJASU_1, FJASU_3, FJASU_4), mid left to right panel (FJASU_12, FJASU_13, FJASU_14, FJASU_15), Bottom left to right panel (FJASU_11, FJASU_16, FJASU_17, FJASU_18).

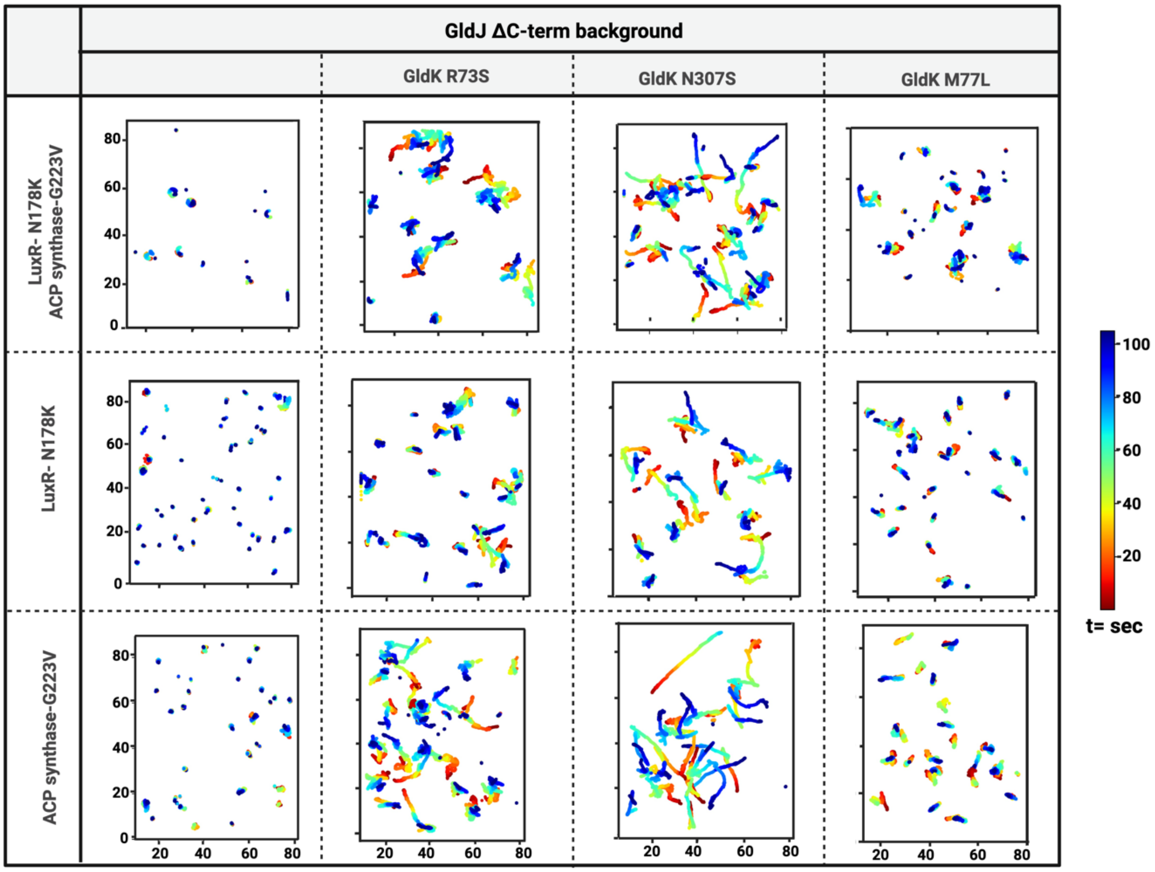

Figure S3. Microscopic imaging and single cell tracking over a glass surface provided further evidence that G223V mutation in ACP synthase and N178K mutation in a LuxR-like protein do not play a role in the restoration of motility. The images show individual cell trajectories, color-coded by time (seconds). Strain list from top left to right panel (FJASU_54, FJASU_1, FJASU_3, FJASU_4), mid left to right panel (FJASU_12, FJASU_13, FJASU_14, FJASU_15), bottom left to right panel (FJASU_11, FJASU_16, FJASU_17, FJASU_18).

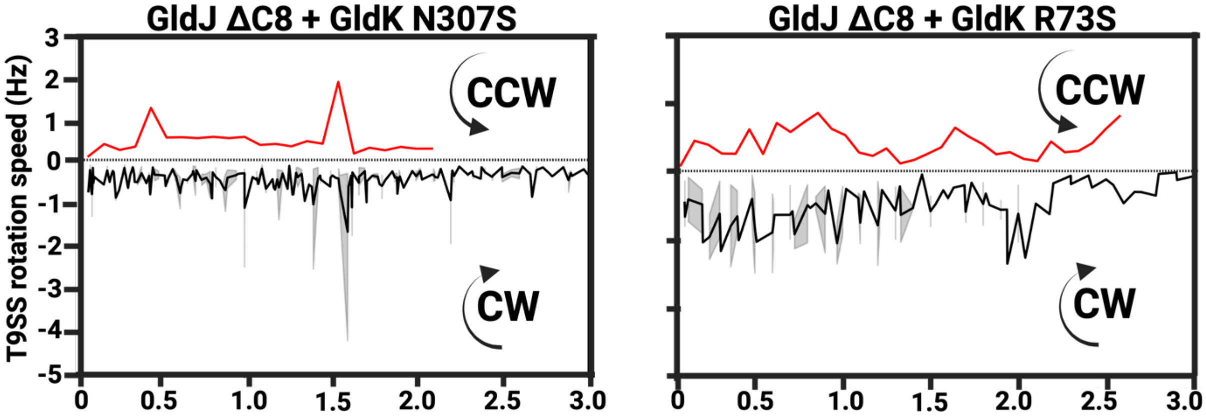

Figure S4. The rotational direction of T9SS motors of evolved GldJ ∆C8 strains with either GldK N307S (FJASU_19, n=17) or GldK R73S (FJASU_21, n=6) mutation, rotate predominately in CW direction, with mean speeds of 0.34 Hz and 0.56 Hz, respectively. Unlike the parent GldJ ΔC8 strain where all T9SS motors rotate CW, a few CCW motors (red line) are observed in the evolved strain. The (+, -) signs indicate CCW and CW rotational directions, respectively.

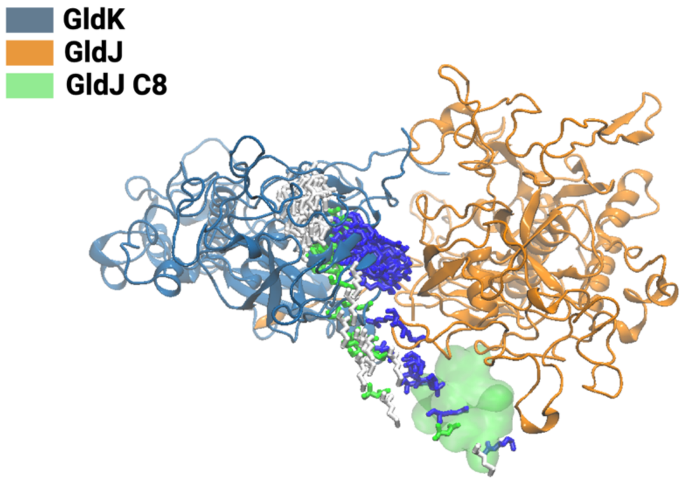

Figure S5. MD stimulations show that in the GldJ ΔC8 strain, the functional GldK residues identified by the suppressor screen (blue, white, and green stick diagrams) can dynamically compensate for the region previously occupied by C8 of wild-type GldJ (shown as a green surface). The MD simulations provide further support for the restored motility observed in the evolved strains, as confirmed by experimental data.

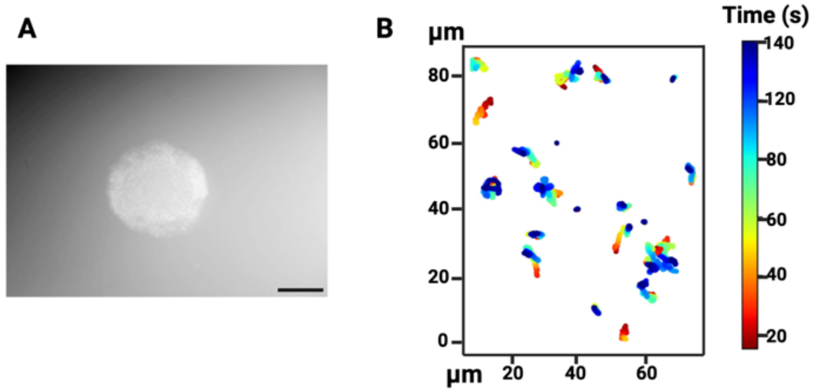

Figure S6. Combination of all three GldK point mutations (GldK R73S, M77L, and N307S) in a GldJ ΔC8 background did not restore **(A)** swarming on agar and **(B)** Single cell-motility over a glass surface. Scale bar for panel A is 6 mm. The images in panel B show individual cell trajectories, color-coded by time (seconds).

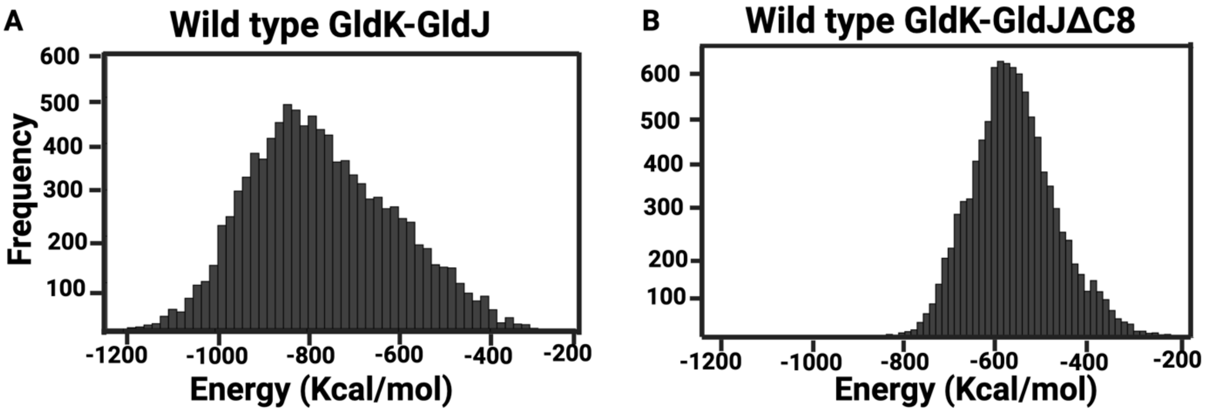

Figure S7. (A) Nonbonded interaction energy analysis illustrating a stronger interaction between GldJ and GldK in the wild-type GldJ and GldK system (-776.4 Kcal/mol average) compared to, **(B)** a weaker interaction in the GldJΔC8 and GldK system (-537.3 Kcal/mol average).

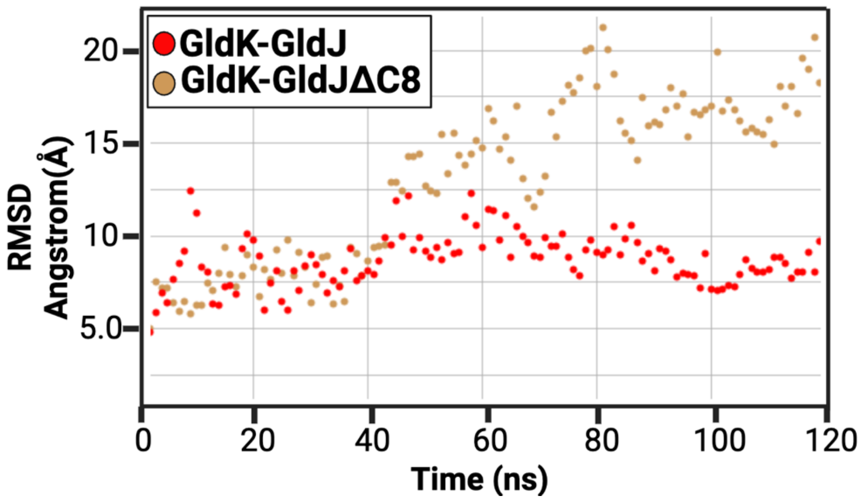

Figure S8. RMSD analysis of GldJ and GldK in both the WT and mutant systems. GldK deviates significantly from its initial structure in the GldJ ΔC8 model reaching a final RMSD of ~20 Å, which is around 10 Å larger than the wild-type model.

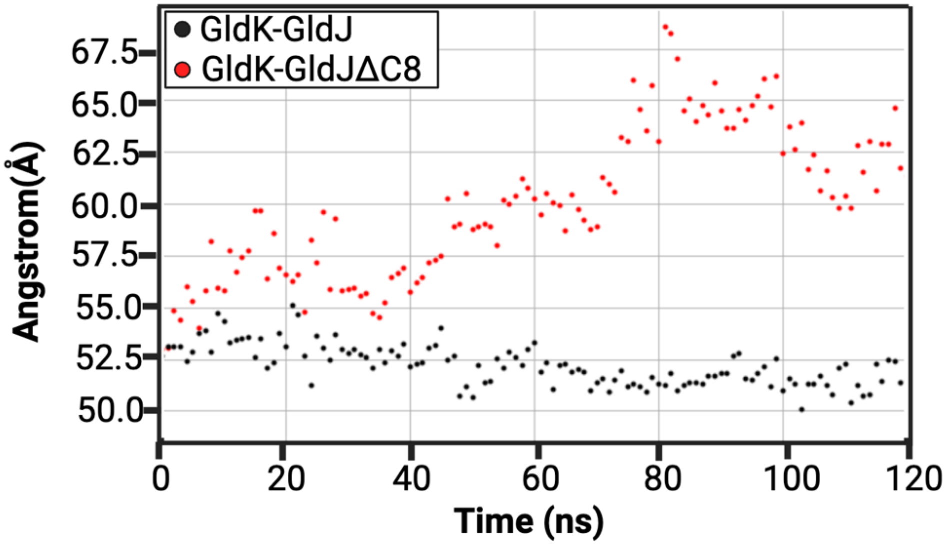

Figure S9. The inter center of mass distances, including all heavy atoms, between GldK and GldJ in the wild-type and GldJ ΔC8 systems recorded periodically throughout the MD simulation.

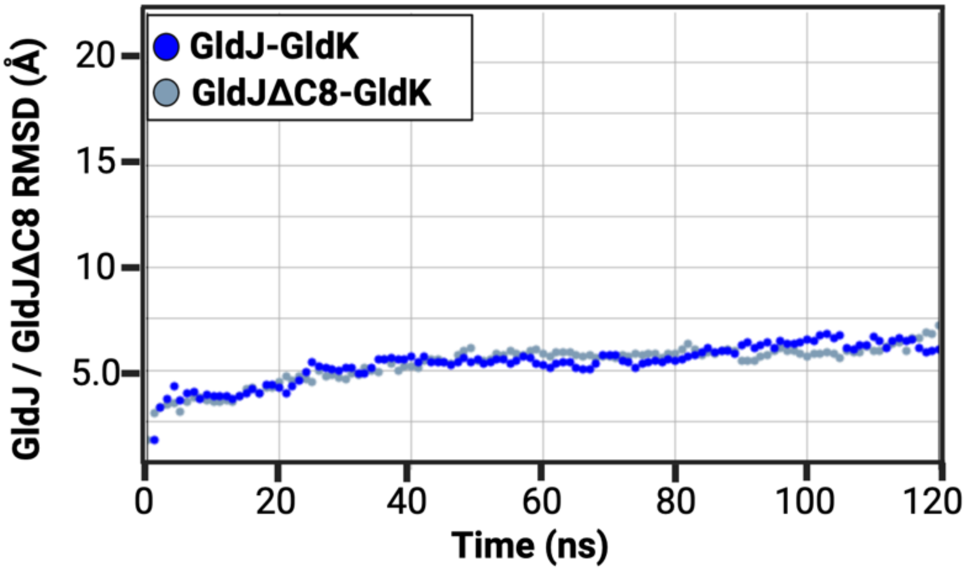

Figure S10. The RMSD of GldJ in the GldJ-GldK system, compared to GldJΔC8 in the GldJΔC8-GldK system, indicates that GldJ does not expand in a manner similar to GldK when C8 of GldJ is deleted.

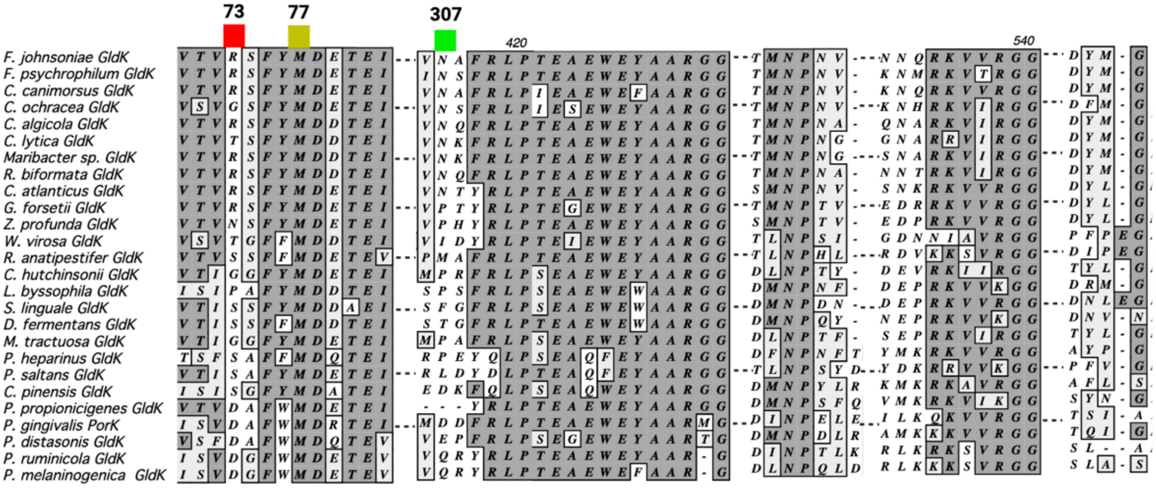

Figure S11. Multiple sequence alignment (MSA) of GldK protein, highlighting point mutations in moderately conserved regions across various species. Each mutation is indicated by a specific color, Red GldKR73S, Yellow GldK M77L and Green GldK N307S. These residues are evolutionarily conserved across species, suggesting their importance in protein function.

SUPPLEMENTARY TEXT

Single amino acid substitutions of LuxR and Acyl Carrier Protein (ACP) Synthase in a GldJ ΔC8 background does not improve motility. The three GldK suppressor strains isolated from our screen also had a N178K mutation in a putative LuxR-like protein (*Fjoh_4220*) and a G223V mutation in an Acyl Carrier Protein (ACP) Synthase (*Fjoh_3810*). No reports suggest that these two proteins influence Bacteroidetes gliding motility, and it's plausible that these mutations were already present in the parent strain prior to the suppressor screen. To gain a deeper understand of their role, individual strains that carry LuxR N178K, ACP G223V, and LuxR N178K plus ACP G223V mutation in a GldJ ΔC-terminal background were generated. When comparing the new strains with mutations in LuxR and ACP to the original GldJ ΔC-terminal strain, there were no noticeable improvements in swarming and single-cell motility (**Supplementary video 5, 6**) The strains that had both GldK R73S, M77L, and N307S mutations, along with the LuxR and ACP mutations, showed similar swarming and single-cell motility characteristics as the strains with only GldK R73S, M77L, and N307S mutations in a GldJ ΔC-terminal background. Similarly, for LuxR N178K and ACP G223V individually GldJ ΔC-terminal background exhibited no motility. Furthermore, when both LuxR N178K and ACP G223V were present in individual GldK point mutants no improvement in speed was observed (**Supplementary video 7, 8**). These results indicate that the suppressor phenotype is not influenced by the LuxR N178K and ACP G223V mutations (**Figs. S3, S4**).

Combinatorial mutagenesis of GldK point mutations in a GldJ ΔC-terminal background does not restore motility. A triple GldK mutant strain, which includes the

mutations GldK R73S, M77L, and N307S in the GldJ ΔC8 background, was created. However, this mutant strain did not exhibit swarming on agar, and its single cells displayed sluggish back and forth motion (**Fig. S6**). These characteristics were comparable to the behavior observed in cells of the original GldJ ΔC8 strain.

SUPPLEMENTARY VIDEOS

Supplementary video 1. Timelapse images of a tethered wild-type cell with a counterclockwise rotating T9SS.

Supplementary video 2. Timelapse images of a tethered GldJ ΔC8 cell with a clockwise rotating T9SS.

Supplementary video 3. Timelapse images of cells containing GldK R73S, GldK M77L and GldK N307S individual point mutations in GldJ ΔC8 background over a glass surface.

Supplementary video 4. Timelapse TIRF images of immunofluorescent SprB motion on the surface of cephalexin treated elongated cells with the following genotypes: wild-type, GldJ ΔC8, and GldK N307S in GldJ ΔC8 background.

Supplementary video 5. Timelapse images, over a glass surface, of GldJ ΔC8 cells containing GldK R73S ACP G223V, GldK M77L ACP G223V, and GldKN307S ACP G223V.

Supplementary video 6. Timelapse images, over a glass surface, of GldJ ΔC8 cells containing GldK R73S LuxR N178K, GldK M77L LuxR N178K, and GldKN307S LuxR N178K.

Supplementary video 7. Timelapse images, over a glass surface, of cells containing ACP_G223V and LuxR_N178K mutation individually a GldJ ∆C8 background and ACP_G223V and LuxR_N178K together in a GldJ ∆C8 background.

Supplementary video 8. Timelapse images, over a glass surface, of cells containing (i) GldK R73S, ACPG223V, and LuxR N178K mutations in a GldJ ∆C8 background. (ii) GldK R73S, ACP G223V, and LuxR N178K mutations in a GldJ ∆C8 background. (iii) GldK N307S, ACP G223V, and LuxR N178K mutations in a GldJ ∆C8 background.

Supplementary video 9. Time-lapse images from tethered cell assays of the evolved GldK mutant strains in a GldJ ΔC8 background, illustrating examples with both CW and CCW rotating T9SS.
